# Optimizing terminology for pancreatectomy: Introducing a new notation system

**DOI:** 10.1002/jhbp.12065

**Published:** 2024-08-11

**Authors:** Kei Yamane, Kazuyuki Nagai, Takayuki Anazawa, Yosuke Kasai, Tomoaki Yoh, Satoshi Ogiso, Yoichiro Uchida, Takashi Ito, Takamichi Ishii, Etsuro Hatano

**Affiliations:** ^1^ Division of Hepato‐Biliary‐Pancreatic Surgery and Transplantation, Department of Surgery, Graduate School of Medicine Kyoto University Kyoto Japan

**Keywords:** nomenclature, notation, pancreas, pancreatectomy, terminology

## Abstract

We introduce a novel notation system for pancreatectomy designed to provide a clear and concise representation of surgical procedures. As surgical techniques and the scope of pancreatic surgeries continue to diversify, existing communication methods among medical professionals regarding the specifics of the surgeries have proven inadequate. Our proposed notation system clearly indicates the approach (open, laparoscopic, or robot‐assisted), type of surgery (e.g., pancreatoduodenectomy, distal pancreatectomy), and extent of resection and accompanying resected organs or vasculature. These elements are all recorded in this order by using abbreviations. For example, a pancreatoduodenectomy with pancreatic transection just above the SMA and combined resection of the SMV would be noted as “OPD(hb')‐SMV”. This new notation system allows for concise expression of the essential information on performed procedures of pancreatic resection, leading to smooth information sharing. This initiative is an essential step towards standardizing pancreatic surgery documentation on a global scale. Here, we present the development and application of this system, highlighting its potential to transform surgical communication and documentation.

## Graphical abstract

Yamane and colleagues introduce a novel notation system for pancreatectomy that provides a clear and concise method for documenting surgical procedures. Using abbreviations, the system specifies the approach, procedures, extent of resection, and concomitantly resected organs and/or vasculature. This systematized notation enhances communication and documentation, promoting global standardization of pancreatectomy terminology.

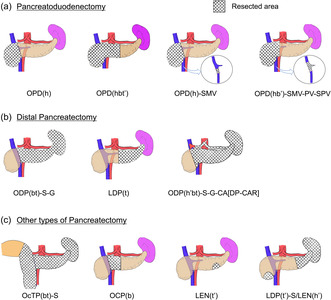

Recent advances in technology and accumulation of knowledge have led to the proliferation of pancreatic resection surgeries worldwide. In addition to conventional open pancreatectomy, minimally invasive pancreatic surgeries, such as laparoscopic or robotic pancreatectomies, have recently been increasing.^1^ Based on evidence of the oncological outcomes, minimally invasive pancreatic surgeries have been applied not only for benign or borderline malignant diseases but also for pancreatic cancer.^2^ However, many hepato‐pancreato‐biliary surgeons still consider that open surgery is preferable to pancreatoduodenectomy (PD) in procedures involving portal vein/superior mesenteric vein resection and arterial resection.^3^ Therefore, all of these approaches will continue to be used for pancreatic resection. Moreover, the extent of pancreatic resection is often dictated by the specific type of pancreatic disease. For example, in the case of pancreatic body or tail cancers, surgical procedures aim to achieve adequate tumor margins, including lymphadenectomy, by completely removing the pancreatic body, tail, and spleen, typically by dissection at the level of the portal vein. However, for borderline malignant or benign tumors of the pancreatic tail, surgical strategies often prioritize the preservation of the pancreatic parenchyma and spleen.^4^ Consequently, pancreatic surgeries have diversified not only in terms of surgical approaches but also in the extent of resection. However, despite the differences in the extent of pancreatic resection involved in these procedures, they are often labeled under the same terminology, such as “distal pancreatectomy (with/without splenectomy).” In pancreatectomies, the extent of resection significantly influences the patient's postoperative endocrine and exocrine functions.^5^ Given the recent improvements in the long‐term prognosis after pancreatic resection, it is crucial for physicians to be well‐informed about the approach and extent of resection to understand and manage the patient's postoperative nutritional status. This knowledge is essential for optimizing patient care and outcomes.

Nagino et al.^6^ proposed the “New World” terminology, a concise and clear notation system, for hepatectomy. This system allows for the simple conveyance of surgical procedures in detail and enhances the understanding of the post‐surgical condition, potentially facilitating communication among medical professionals. Although the terminology was developed based on Couinaud's hepatic segmental anatomy, there is no worldwide definition of the parts of the pancreas. Consequently, it is challenging to create a clear and concise notation system for pancreatectomies. We believe that there is a need for a notation system that clearly defines the approach of pancreatectomy and the parts of the resected pancreas. Such a convenient notation system would greatly assist routine clinical practice. Consequently, we created a new notation system for pancreatic resection.

The general rules of this new notation system (Figure 1a).

1. A prefix indicating the surgical approach is employed, with “O” for open, “L” for laparoscopic, and “R” for robot‐assisted surgeries. In conversion cases, the initial approach method should be recorded as a prefix, followed by a right arrow, and then the final surgical approach method should be described (e.g., L → ODP(b't)‐S).
2. The type of surgical procedure is then indicated: “PD” for pancreatoduodenectomy, “DP” for distal pancreatectomy or left‐sided pancreatectomy, “TP” for total pancreatectomy, “CP” for central pancreatectomy, “EN” for enucleation of pancreatic tumors, “PP” for partial pancreatectomy, and “cTP” for completion total pancreatectomy (Table 1).
3. The extent of the pancreatic resection is denoted by the specific parts removed (head, h; body, b; tail, t) as defined by Classification of Pancreatic Carcinoma fourth English edition by the Japan Pancreas Society.^7^ In other words, pancreas head is defined as the area from the anterior to the SMV to its right side, the body of the pancreas extends from the left edge of the SMV to the left edge of the aorta, and the tail extends from the left edge of the aorta to the left side of the pancreas (Figure 1b). An apostrophe (') is added when the resection is partial. The resected regions are enclosed in parentheses. For a completion total pancreatectomy (cTP), the surgical procedure corresponding to the remaining pancreatic extent is noted. For example, an open completion total pancreatectomy following a pancreatoduodenectomy (PD) would be notated as “OcTP(bt)‐S”, and an open completion pancreatectomy following a distal pancreatectomy (DP) would be notated as “OcTP(h)”.
4. Organs and vessels resected en bloc beyond the procedural nature are noted in order—organs, veins (portal vein system), and arteries—connected by a hyphen ‘‐’. For “PD” gallbladder, distal bile duct, and duodenum are not added, and vasculature that is typically resected in standard procedures (e.g., the gastroduodenal artery in PD, the splenic artery/vein in DP) is not considered as organs involved in combined resection. Organs resected separately, not en bloc, are listed after a slash ‘/’. Table 2 shows the notations of combined resection; other organs (“AC” for ascending colon, “AD” for adrenal grand, “DC” for descending colon, “H” for liver, “KD” for kidney, “S” for spleen, “ST” for stomach, “TC” for transverse colon) and blood vessels (“CA” for celiac axis, “CHA” for common hepatic artery, “GDA” for gastroduodenal artery, “IVC” for inferior vena cava, “PV” for portal vein, “SMA” for superior mesenteric artery, “SMV” for superior mesenteric vein, “SPA” for splenic artery, “SPV” for splenic vein). In combined liver resection, the resected segments are documented according to the notation used for liver resection.^6^ The extent of lymphadenectomy was not included in this study.
5. Abbreviations widely recognized, such as DP‐CAR (distal pancreatectomy with celiac axis resection), PD‐SAR (pancreaticoduodenectomy with splenic artery resection), and Kimura procedure (splenic artery and vein preserved DP), may provide clearer understanding of the procedures than our notation system. Therefore, it is acceptable to list them with brackets [] at the end. Table 3 and Figure 2 show examples of new notations for various types of pancreatectomies.

**FIGURE 1:**
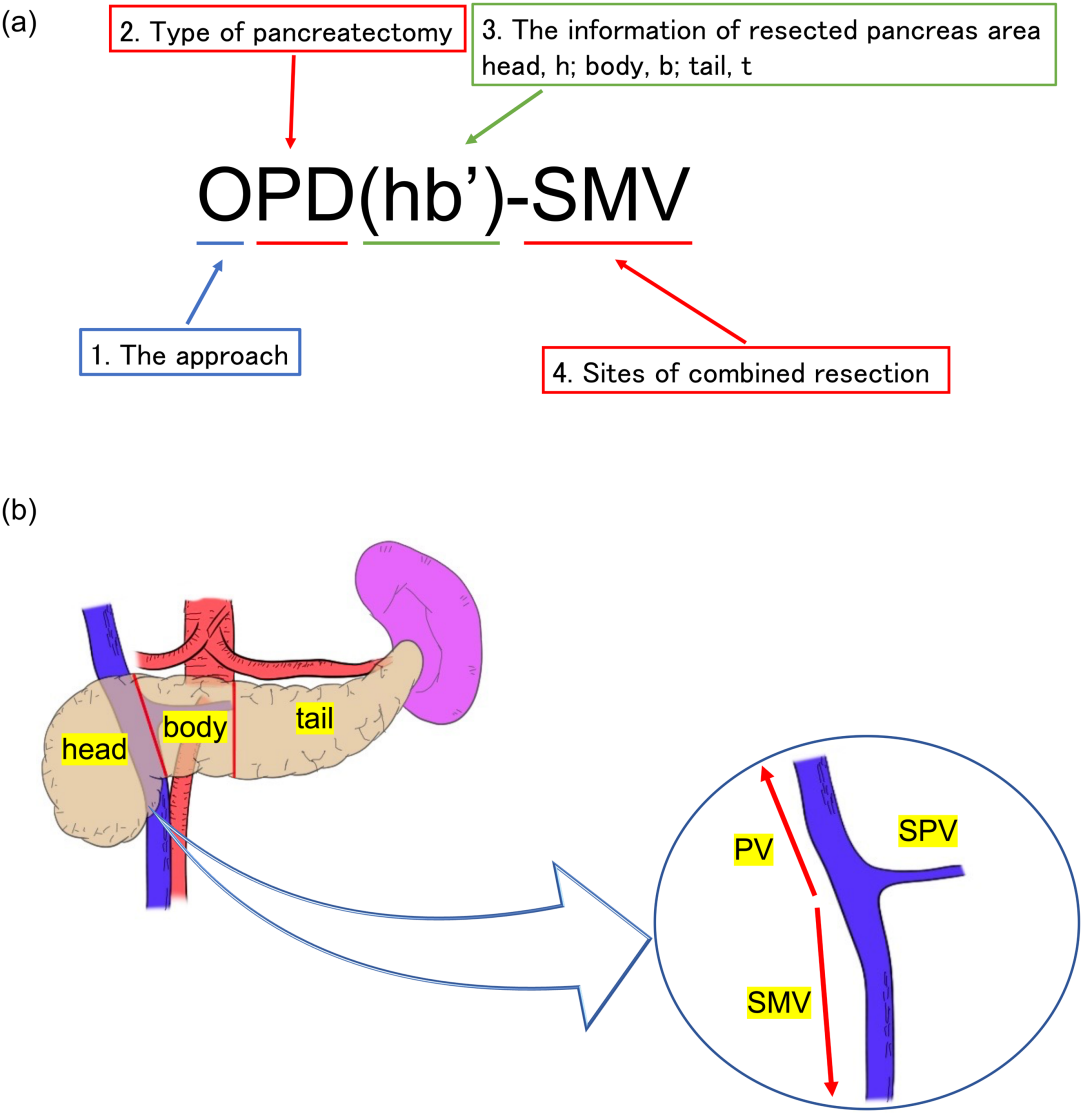
(a) The rules of notation for pancreatectomy in Kyoto University. (b) The definitions of the pancreas segments and portal vein system. The boundary between the pancreatic head and the body is at the level of the left edge of the portal vein, while the boundary between the pancreatic body and tail is at the level of the left edge of the aorta. The portal vein is defined as the section from where the superior mesenteric vein (SMV) and splenic vein (SPV) converge to the porta hepatis.

**TABLE 1:** Abbreviations for pancreatectomy.

Pancreatectomy	Abbreviations
Pancreatoduodenectomy	PD
Distal pancreatectomy	DP
Total pancreatectomy	TP
Central pancreatectomy	CP
Enucleation	EN
Partial pancreatectomy	PP
Completion total pancreatectomy	cTP

**TABLE 2:** Notations for combined resection.

Organs	Notation	Vessels	Notation
Ascending colon	AC	Celiac axis	CA
Adrenal gland	AG	Common hepatic artery	CHA
Bile duct	B	Gastroduodenal artery	GDA
Descending colon	DC	Inferior vena cava	IVC
Duodenum	DU	Portal vein	PV
Gerota's fascia	G	Superior mesentric artery	SMA
Gallbladder	GB	Superior mesentric vein	SMV
Liver	H	Splenic artery	SPA
Kidney	KD	Splenic vein	SPV
Spleen	S		
Stomach	ST		
Transverse colon	TC		

**TABLE 3:** Examples of the new notations for pancreatectomy.

Surgical procedures	Pancreas transection line (resected pancreas area)	New notation
Open PD	SMV (head)	OPD(h)
Open PD with SMV, PV, and SPV resection	SMA (head, part of body)	OPD(hb')‐SMV‐PV‐SPV
Open PD with SPA resection (i.e., PD‐SAR)	Left side of aorta (head, body, part of tail)	OPD(hbt')‐SPA[PD‐SAR]
Laparoscopic PD	SMA (head, part of body)	LPD(hb')
Laparoscopic PD converted to open procedure	SMA (head, part of body)	L → OPD(hb')
Robotic PD	SMV (head)	RPD(h)
Open duodenum‐preserving total pancreatic head resection (i.e., DPPHR) with bile duct resection	SMV (head)	OPP(h)‐B
Open DP with splenectomy and left adrenalectomy	SMV (body, tail)	ODP(bt)‐S‐AG‐G
Open DP with CA resection (i.e., DP‐CAR) and cholecystectomy	Under GDA (part of head, body, tail)	ODP(h'bt)‐S‐G‐CA/GB[DP‐CAR]
Open DP with splenectomy and SMV resection and partial hepatectomy (S2)	SMV (body, tail)	ODP(bt)‐S‐G‐SMV/H2'
Laparoscopic spleen‐preserving DP (Kimura procedure)	Left side of aorta (tail)	LDP(t) [Kimura]
Laparoscopic spleen‐preserving DP (Warshaw procedure)	SMA (part of body, tail)	LDP(b't)‐SPA‐SPV [Warshaw]
Robotic DP with splenectomy	SMA (part of body, tail)	RDP(b't)‐S
Laparoscopic DP with splenectomy and enucleation of pancreas head tumor	SMV (body, tail), in pancreas head	LDP(bt)‐S/LEN(h)
Open TP with splenectomy	Absent (head, body, tail)	OTP(hbt)‐S
Open CP	SMV, left edge of aorta (body)	OCP(b)
Laparoscopic enucleation	in pancreas head (part of head)	LEN(h')
Open completion TP with splenectomy after PD	Absent (body, tail)	OcTP(bt)‐S

Abbreviations: AD, adrenal grand; CA, celiac axis; CHAR, common hepatic artery resection; CP, central pancreatectomy; DP, distal pancreatectomy; GB, gallbladder; GDA, gastroduodenal artery; H, liver; PD, pancreatoduodenectomy; PV, portal vein; S, spleen; SAR, splenic artery resection; SMA, superior mesenteric artery; SMV, superior mesenteric vein; SPV, splenic vein; TP, total pancreatectomy.

**FIGURE 2:**
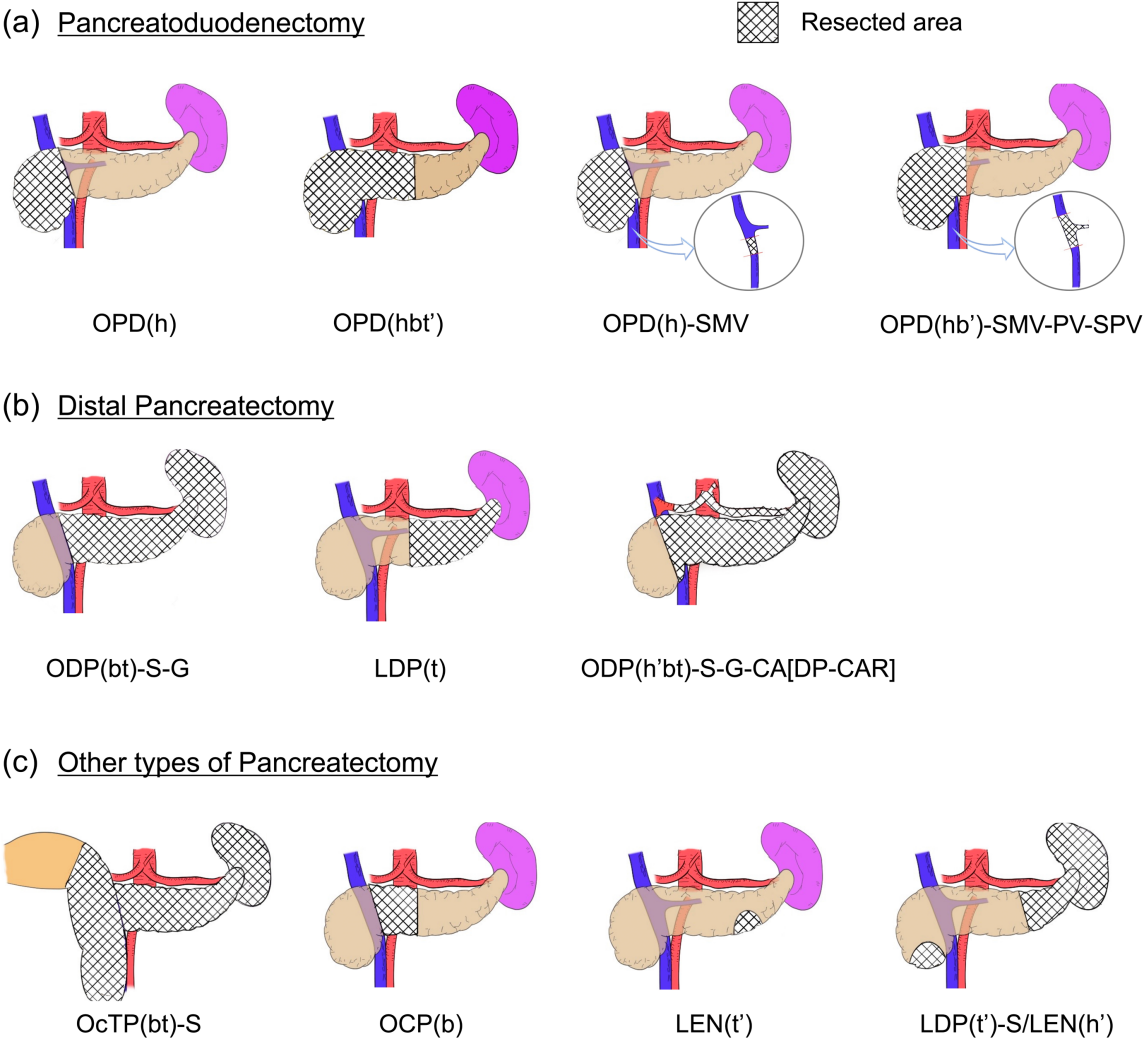
Diagrams and new notations for different pancreatectomy procedures. The resected area is indicated with hatching. (a) Pancreatoduodenectomy. (b) Distal pancreatectomy. (c) Other types of pancreatectomy.

This new notation system focuses on the extent of pancreatic resection and does not reflect the surgical procedure. For example, the distal pancreatectomy with resection of the spleen, Gerota's fascia, and left adrenal gland for pancreatic tail cancer is expressed as “ODP(bt)‐S‐AG‐G” in this notation regardless of whether the surgical procedure is a “radical antegrade modular pancreatosplenectomy (RAMPS)”^8^ or a “left‐posterior approach”.^9^ Surgical procedures vary by institution, and including them would make the notation too wordy, which goes against our goal of clear and concise terminology. We believe that surgical procedures should be looked up in the surgical records.

This notation system has some limitations that the authors have carefully discussed. First, the range of stomach resection is not specified. In recent years, the range of gastric resection in PD has typically involved pylorus‐preserving PD or subtotal stomach‐preserving PD, which are selected based on the surgeon's preference or institution policy. Subtotal stomach‐preserving PD may result in fewer instances of delayed gastric emptying compared with pylorus‐preserving PD, and we consider that the extent of stomach resection does not significantly impact long‐term nutritional outcomes.^10^ Therefore, it was not included in this notation system. Our new notation system aims to express the extent of resection or excision rather than preservation. Therefore, it lacks a mechanism to explicitly denote preservation when structures such as the duodenum, pylorus, and splenic vessels, which are typically omitted in notation rules for combined resection, are actually preserved. Second, information about reconstructions after pancreatectomy is also not included in this notation. This might impact the long‐term nutritional status and represents a critical factor indicating the state of the abdominal cavity post‐surgery. However, since some pancreatectomies (e.g., DP) do not require reconstruction, and including details of the reconstruction would make the notation complicated and harder to understand, it was decided that information about reconstructions should not be included. When information on the state of the abdominal cavity, such as the reconstruction methods, is clinically required, surgical records or figures should be reviewed. Finally, this notation system does not include information on additional pancreatic resections, which may be performed if malignant findings are detected at the pancreatic margin. While the presence of additional resections is crucial information in pancreatectomy, this notation system focuses simply on showing the final extent of the pancreas that was removed. Accordingly, it does not incorporate such details.

Recently, a new terminology for DP has been proposed by consensus among experts in pancreatic surgery, which classifies the scope and site of resection into subcategories that are more clearly defined.^11^ Thus, the need for new terminology for pancreatectomy has been recognized by pancreatic surgeons worldwide. Our new notation system for pancreatectomy was designed to provide a simple and comprehensible representation of the surgical procedures. As the approaches and extents of pancreatectomies diversify, adopting this notation system is desirable to avoid confusion among medical professionals regarding the specifics of the surgeries performed on each patient. Furthermore, this notation system may be useful for examining the factors associated with short and/or long‐term complications of pancreatic resection surgeries. At Kyoto University Hospital, we have already used this notation method, which has proven to be highly beneficial in enhancing information sharing among medical staff during clinical practice and conferences. We hope that this notation system, which supports the sharing of information on pancreatic resection, will be widely used.

## CONFLICT OF INTEREST STATEMENT

The authors declare no conflicts of interest in association with the present study.
